# PLSCR1 Regulates the Physiology of Fibroblast‐Like Synoviocytes via Modulating the STAT1 Signaling Pathway

**DOI:** 10.1002/iid3.70294

**Published:** 2025-10-27

**Authors:** Tianhua Chen, Jiaojiao Wang

**Affiliations:** ^1^ Department of Pain Management, The Sixth Hospital of Wuhan Affiliated Hospital of Jianghan University Wuhan China; ^2^ Public Relations Department Hubei Provincial Clinical Laboratory Center Wuhan China

**Keywords:** fibroblast‐like synoviocytes, PLSCR1, rheumatoid arthritis, STAT1

## Abstract

**Background:**

Rheumatoid arthritis (RA) is a chronic autoimmune disease characterized by synovial inflammation and joint destruction. Elucidating the molecular mechanisms underlying the pathogenesis is crucial for identifying novel therapeutic targets. Phospholipid scramblase 1 (PLSCR1) has been implicated in systemic autoimmune diseases. However, the function and underlying mechanism in RA remains unclear.

**Methods:**

Reverse transcriptase‐quantitative polymerase chain reaction was used to detect the expression levels of PLSCR1 in the serum of 30 RA patients and 30 healthy controls. The function of PLSCR1 in human fibroblast‐like synoviocytes (HFLSs) was investigated by siRNA‐mediated knockdown. Cell proliferation, apoptosis, and inflammatory cytokine production were assessed through 5‐ethynyl‐2'‐deoxyuridine (EdU) assays, flow cytometry and enzyme‐linked immunosorbent assays. The regulatory relationship between PLSCR1 and signal transducer and activator of transcription 1 (STAT1) was further explored using 2‐NP rescue experiments.

**Results:**

PLSCR1 was significantly upregulated in the serum of RA patients. Silencing PLSCR1 in HFLSs led to decreased proliferation, increased apoptosis, reduced levels of tumor necrosis factor alpha, interleukin‐1‐beta, and IL‐6, and downregulation of STAT1 expression. Notably, activation of STAT1 signaling reversed the effects of PLSCR1 knockdown, restoring proliferative capacity and inflammatory cytokine production while reducing apoptosis.

**Conclusion:**

PLSCR1 is upregulated in RA and regulates the proliferation, apoptosis and inflammation of FLSs by modulating the STAT1 signaling pathway. These findings suggest that PLSCR1 may serve as a potential molecular target for RA therapy.

## 1 Introduction

Rheumatoid arthritis (RA) is a chronic systemic autoimmune disease triggered by a combination of genetic and environmental factors, with a global prevalence of approximately 0.5%–1% [1]. It is characterized by synovial inflammation and progressive destruction of articular cartilage and bone, ultimately leading to joint deformities and functional impairment [2]. Although current disease‐modifying antirheumatic drugs (DMARDs) have significantly improved clinical outcomes [3], a considerable proportion of patients fail to achieve sustained remission, highlighting the urgent need to identify novel molecular targets for therapy.

Synovitis is a central pathological feature of RA, closely associated with autoimmune activation [4]. Autoantibody production and inflammatory pathway activation can occur years before the onset of clinical symptoms [5]. In the inflamed synovial microenvironment of RA, fibroblast‐like synoviocytes (FLSs) undergo aberrant activation and acquire a tumor‐like phenotype, characterized by sustained proliferation, resistance to apoptosis, and enhanced migratory and invasive capabilities [5, 6, 7]. Activated FLSs contribute to disease progression by continuously producing pro‐inflammatory cytokines, chemokines, and matrix metalloproteinases, thereby driving local inflammation and joint destruction [8, 9]. Consequently, FLSs are increasingly recognized as critical therapeutic targets in RA [10, 11]. Pharmacological inhibition of FLS‐dependent effector molecules might be a promising option for FLS‐targeted therapy in RA [12].

Phospholipid scramblase 1 (PLSCR1) is the most widely studied member of the PLSCR family [13]. The human PLSCR1 gene is located on chromosome 3 and contains nine exons, with the coding region located between exons 2 and 9 [14]. PLSCR1 is a typical interferon‐stimulated gene (ISG) that is strongly induced by type I versus type II interferons [15, 16]. Zhou et al. demonstrated that IFN‐α significantly upregulated PLSCR1 expression in various human cell lines, and that the promoter region contained an IFN‐stimulated response element (ISRE), which is essential for interferon‐dependent transcriptional activation [17]. Further studies have shown that IFN‐induced PLSCR1 expression is dependent on signal transducer and activator of transcription (STAT1) activation and requires the synergistic involvement of protein kinase Cδ and the c‐Jun N‐terminal kinase (JNK) pathway [18]. As a calcium‐dependent membrane‐associated protein, PLSCR1 mediates bidirectional translocation of phospholipids across the plasma membrane, and plays crucial roles in apoptosis, phagocytic recognition, and membrane asymmetry maintenance [19, 20, 21]. Notably, the expression level of PLSCR1 was upregulated in several chronic autoimmune diseases, including systemic lupus erythematosus (SLE) [22], suggesting the potential involvement in the regulation of inflammatory responses. However, the specific role of PLSCR1 in RA, particularly whether it contributes to synovitis and joint destruction by modulating FLSs, remains largely unexplored.

Notably, PLSCR1 has been closely linked to the Janus kinase/signal transducer and activator of the transcription (JAK/STAT) signaling pathway [23]. PLSCR1 was found to activate the STAT signaling pathway in breast cancer [24]. Aberrant activation of the STAT pathway has been well documented in the pathogenesis of RA [25]. Studies have shown that activated STAT3 protein inhibitors regulate the migration and invasion of FLSs in RA [26]. Therefore, investigating whether PLSCR1 modulates FLSs behavior through the STAT signaling pathway may offer new insights into the molecular mechanisms underlying RA development.

This study aims to clarify the role and molecular mechanisms of PLSCR1 in RA, potentially offering new insights into RA pathogenesis and identifying novel targets for targeted therapeutic strategies.

## 2 Materials and Methods

### 2.1 Tissue Sample Collection

Serum samples were collected from 30 patients with RA and 30 normal healthy volunteers. All RA patients met the revised classification criteria established by the American College of Rheumatology (ACR) [27]. Patients with other autoimmune diseases, persistent inflammatory diseases, or cancers were excluded in this study. The clinical characteristics of RA patients were presented in Table 1. All samples were obtained from the hospital with approval from the institutional ethics committee (Approval no. JHDXLL2025—009). Written informed consent was obtained from every patient.

**Table 1:** Clinical characteristics of RA patients.

Parameters	RA patients, *N* = 30	Healthy controls, *N* = 30
Male/female	15/15	15/15
Age (years)	45–67	50–66
Disease duration (years)	2–13	—
ESR (mm/h)	43.0–67.0	—
CRP (mg/L)	7.2–69.2	—
RF (U/mL)	488.3–1159.5	—
Anti‐CCP positive, *n* (%)	24 (80)	—
Medicine use, *n* (%)		
DMARDs	25 (83.3)	—
NSAIDs	8 (26.7%)	—

Abbreviations: anti‐CCP, antibodies to cyclic citrullinated peptides; CRP, C‐reactive protein; DMARDs, disease‐modifying antirheumatic drugs; ESR, erythrocyte sedimentation rate; NSAIDs, non‐steroidal anti‐inflammatory drug; RF, rheumatoid factor.

### 2.2 Cell Culture

Human fibroblast‐like synoviocytes (HFLSs) from human normal joint synovial tissue were obtained from the Cellverse Co. Ltd. (HUM‐iCell‐s010). Cells were cultured in Dulbecco's modified eagle medium/F12 medium supplemented with 10% fetal bovine serum and 1% penicillin–streptomycin, and maintained at 37°C in a humidified incubator with 5% CO₂.

### 2.3 Cell Transfection and Drug Treatment

Control‐small interfering RNA (siRNA) and PLSCR1‐siRNA were purchased from GenePharma (Shanghai, China). Transfection was performed using Lipofectamine 3000 reagent (Invitrogen, USA) according to the manufacturer's protocol. Briefly, HFLSs were seeded in six‐well plates and transfected when cell confluency reached approximately 70%. Control‐siRNA or PLSCR1‐siRNA was diluted in Opti‐MEM (Gibco, USA), and Lipofectamine 3000 was diluted separately. After incubation at room temperature for 5 min, the two solutions were mixed and allowed to stand for 15 min to form transfection complexes. The mixture was then added to the cell culture medium, and cells were incubated for 48 h before downstream analysis.

The STAT1 enhancer (2‐NP, HY‐W013523) was obtained from MedChemExpress. Following siRNA transfection, cells were treated with the 2‐NP (5 μmol/mL) for 24 h before being harvested for subsequent experiments.

### 2.4 Reverse Transcriptase‐Quantitative Polymerase Chain Reaction (RT‐qPCR)

To extract the total RNA, TRIpure Total RNA Extraction Reagent (ELK Biotechnology) was used according to the manufacturer's instructions. Reverse transcription was carried out using the EntiLink 1st Strand cDNA Synthesis Kit (ELK Biotechnology) to synthesize cDNA. RT‐qPCR was applied on a QuantStudio 6 Flex Real‐Time PCR System (Life Technologies) using EnTurbo SYBR Green PCR SuperMix (ELK Biotechnology). ACTIN was used as the internal control, and relative gene expression levels were calculated using the $2^{-∆∆C_{t}}$ method. All experiments were performed in triplicate to ensure reproducibility. Primer sequences for the target genes are listed in Table 2.

**Table 2:** The RT‐qPCR primer sequences.

Name	Sequence (5′–3′)	
H‐ACTIN	Sense	GTCCACCGCAAATGCTTCTA
	Antisense	TGCTGTCACCTTCACCGTTC
H‐PLSCR1	Sense	CCAAGGACCTCCAGGATATAGTG
	Antisense	CCAGGTGGACAGTTTAATGGAG
H‐STAT1	Sense	ACTTTCCCTGACATCATTCGC
	Antisense	TCTACAGAGCCCACTATCCGAG

### 2.5 Western Blot Analysis Assay

Cells were washed twice with phosphate‐buffered saline (PBS) and lysed on ice for 30 min using radioimmunoprecipitation assay buffer (ASPEN) supplemented with protease inhibitors (Roche). Lysates were centrifuged at 12,000×*g* for 5 min at 4°C, and the supernatant was collected for protein concentration determination using the bicinchoninic acid assay (ASPEN). Equal amounts of protein (40 μg) were separated by sodium dodecyl sulfate‐polyacrylamide gel electrophoresis and transferred to polyvinylidene fluoride membranes (Millipore, USA). Membranes were blocked with 5% nonfat milk in Tris Buffered Saline Tween (TBST) for 1 h at room temperature, followed by incubation with primary antibodies overnight at 4°C. The primary antibodies used were: β‐Actin (BEIJING TDY BIOTECH, TDY051, 1:10,000), PLSCR1 (Abcam, ab191514, 1:1,000), Bcl‐2 (Cell Signaling Technology, 3498, 1:1,000), Bax (Cell Signaling Technology, 2772, 1:2,000), and STAT1 (Proteintech, 82016‐1‐RR, 1:5,000). After washing three times with TBST, membranes were incubated with HRP‐conjugated secondary antibodies (1:5000) for 1 h at room temperature. Finally, signals were visualized using enhanced chemiluminescence and detected with a chemiluminescent imaging system. Band intensities were quantified using AlphaEaseFC software.

### 2.6 EdU Assay

The EdU assay was used to evaluate cell proliferation. Briefly, cells were seeded onto glass coverslips in 6‐well plates and cultured until they reached 70%–80% confluency. According to the experimental design, cells were subjected to transfection or drug treatment. After 48 h, the culture medium was removed, and the cells were washed three times with PBS. Cells were then fixed with 4% paraformaldehyde for 20 min, followed by another three PBS washes. Next, cells were permeabilized with a permeabilization buffer for 10 min at room temperature. 1× EdU‐488 green fluorescent labeling solution was freshly prepared and added to the cells, which were incubated at 37°C for 30 min. After incubation, cells were washed three times with PBS, followed by staining with 1× Hoechst 33342 for 5 min at room temperature in the dark to label nuclei. After a final PBS wash, coverslips were mounted for imaging. EdU‐positive cells were observed and imaged using a fluorescence microscope. The EdU‐positive rate was calculated as the number of EdU‐positive cells divided by the total number of cells × 100%.

### 2.7 Flow Cytometry for Apoptosis Detection

Cells were assayed for apoptosis using the Annexin V‐FITC Apoptosis Detection Kit (BD). Cells were inoculated at 6 × 10⁵ cells/well in 6‐well plates after transfection or drug treatment. Cells were digested using EDTA‐free trypsin and collected by centrifugation at 300 g for 5 min at 4°C. Cells were suspended in 100 µL of 1× Binding Buffer, 5 µL of Annexin V‐FITC and 5 µL of LPI were added, and the plate was incubated for 15 min at room temperature, protected from light. Cells were washed twice with pre‐cooled 1× Binding Buffer, and 500 µL of 1× Binding Buffer was added, mixed, and placed on a flow cytometer (Beckman). The proportion of apoptotic cells was analyzed using FlowJo software (version 10.8.1).

### 2.8 Enzyme‐Linked Immunosorbent Assay (ELISA)

The concentrations of tumor necrosis factor alpha (TNF‐α), interleukin‐1‐beta (IL‐1β), and IL‐6 in cell culture supernatants were measured using ELISA kits (ELK Biotechnology) according to the manufacturer's instructions. Absorbance was recorded at 532 nm using a microplate reader.

### 2.9 Statistical Analysis

All experiments were independently biological repeated at least three times. Data were presented as the mean ± standard deviation (SD). Comparisons between two groups were performed using an unpaired Student's *t*‐test, while differences among multiple groups were assessed by one‐way analysis of variance (ANOVA), followed by Tukey's post hoc test. Statistical analyses were conducted using GraphPad Prism 8.0 software. A *p* value < 0.05 was considered statistically significant.

## 3 Results

### 3.1 PLSCR1 Was Upregulated in the Serum of RA Patients

**Figure 1:**
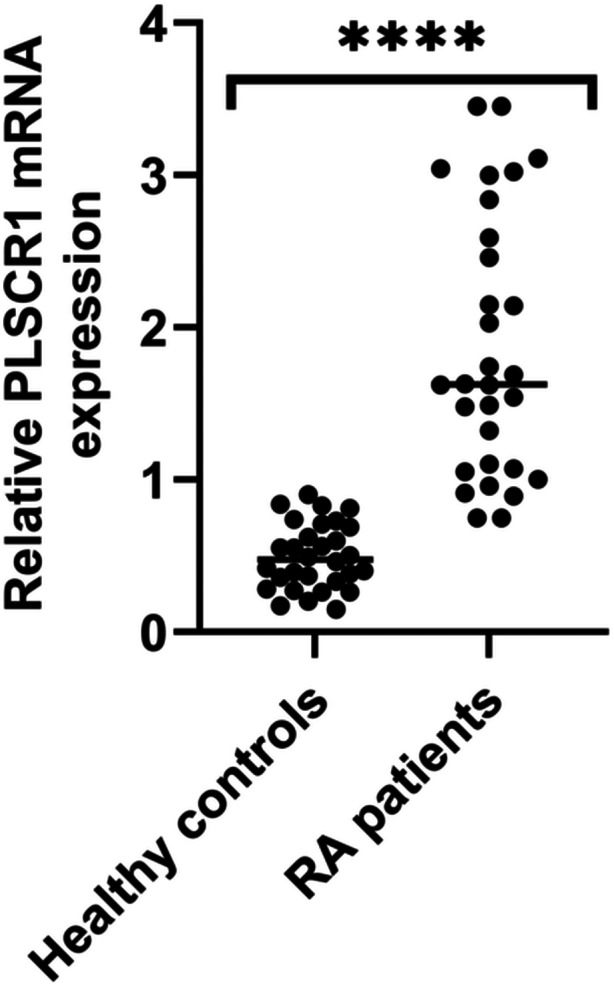
PLSCR1 is upregulated in the serum of RA patients. The mRNA expression of PLSCR1 in serum samples from RA patients and healthy controls measured by RT‐qPCR. Data are presented as mean ± SD; ****p < 0.0001.

To investigate the expression of PLSCR1 in patients with RA, the serum samples were collected from 30 RA patients and 30 healthy volunteers. The mRNA expression levels of PLSCR1 were measured using RT‐qPCR, and the results showed that PLSCR1 expression was significantly higher in the serum of RA patients compared to healthy controls (Figure 1). These findings indicated that PLSCR1 expression was upregulated in RA patients.

### 3.2 The Roles of PLSCR1 in HFLSs

**Figure 2:**
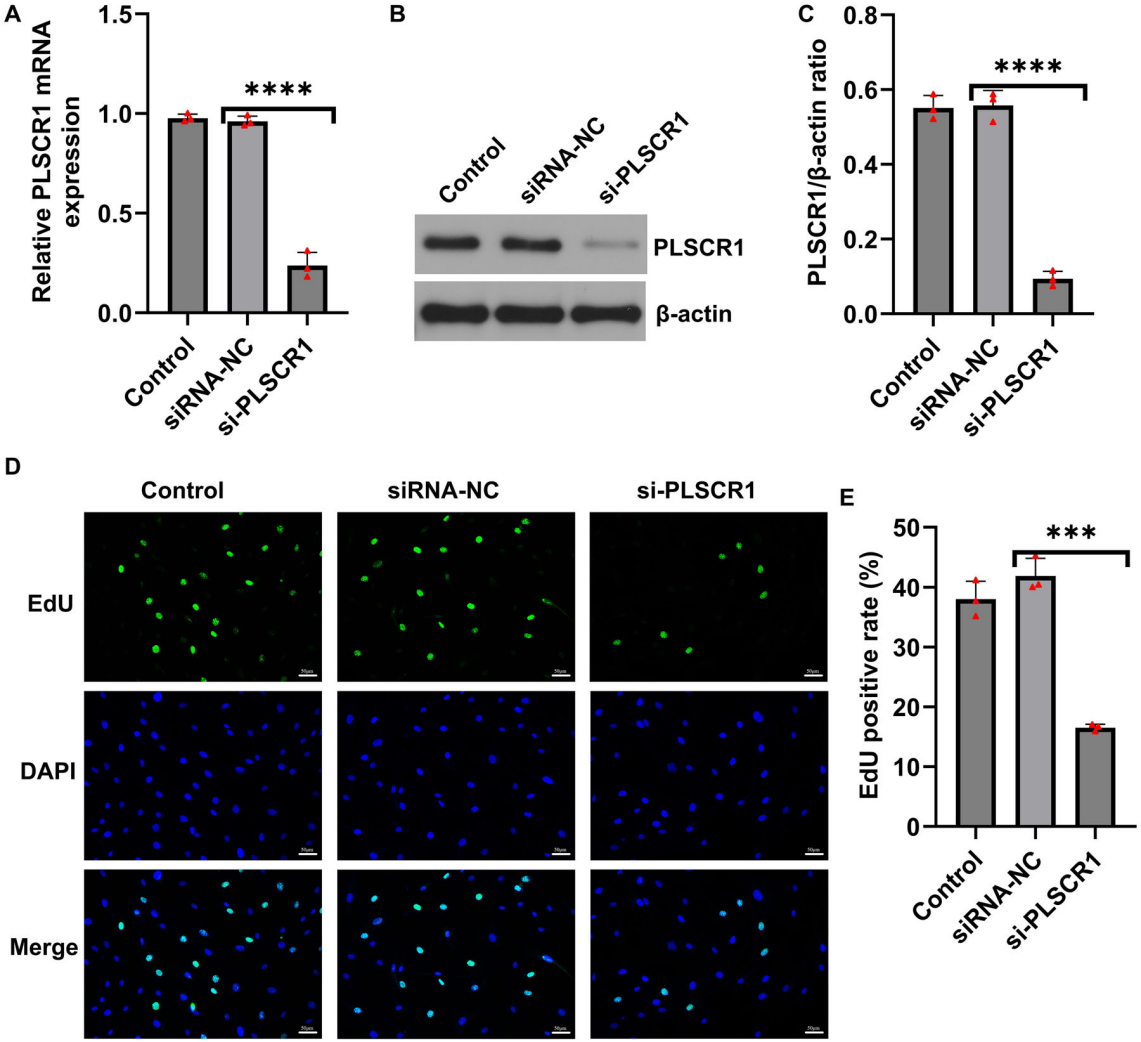
PLSCR1 regulates proliferation in HFLSs. (A) The mRNA level of PLSCR1 in HFLSs detected by RT‐qPCR; (B and C) The protein level of PLSCR1 in HFLSs measured by western blot analysis; (D and E) EdU assay assessed cell proliferation in control and PLSCR1‐silenced HFLSs. Data are presented as mean ± SD; ***p < 0.001; ****p < 0.0001.

To investigate the role of PLSCR1 in HFLSs, we silenced PLSCR1 using siRNA. RT‐qPCR and western blot analysis confirmed that mRNA and protein levels of PLSCR1 were significantly reduced in the PLSCR1‐siRNA group compared to the control‐siRNA group (Figure 2A–C), indicating that the siRNA effectively knocked down the expression of PLSCR1.

**Figure 3:**
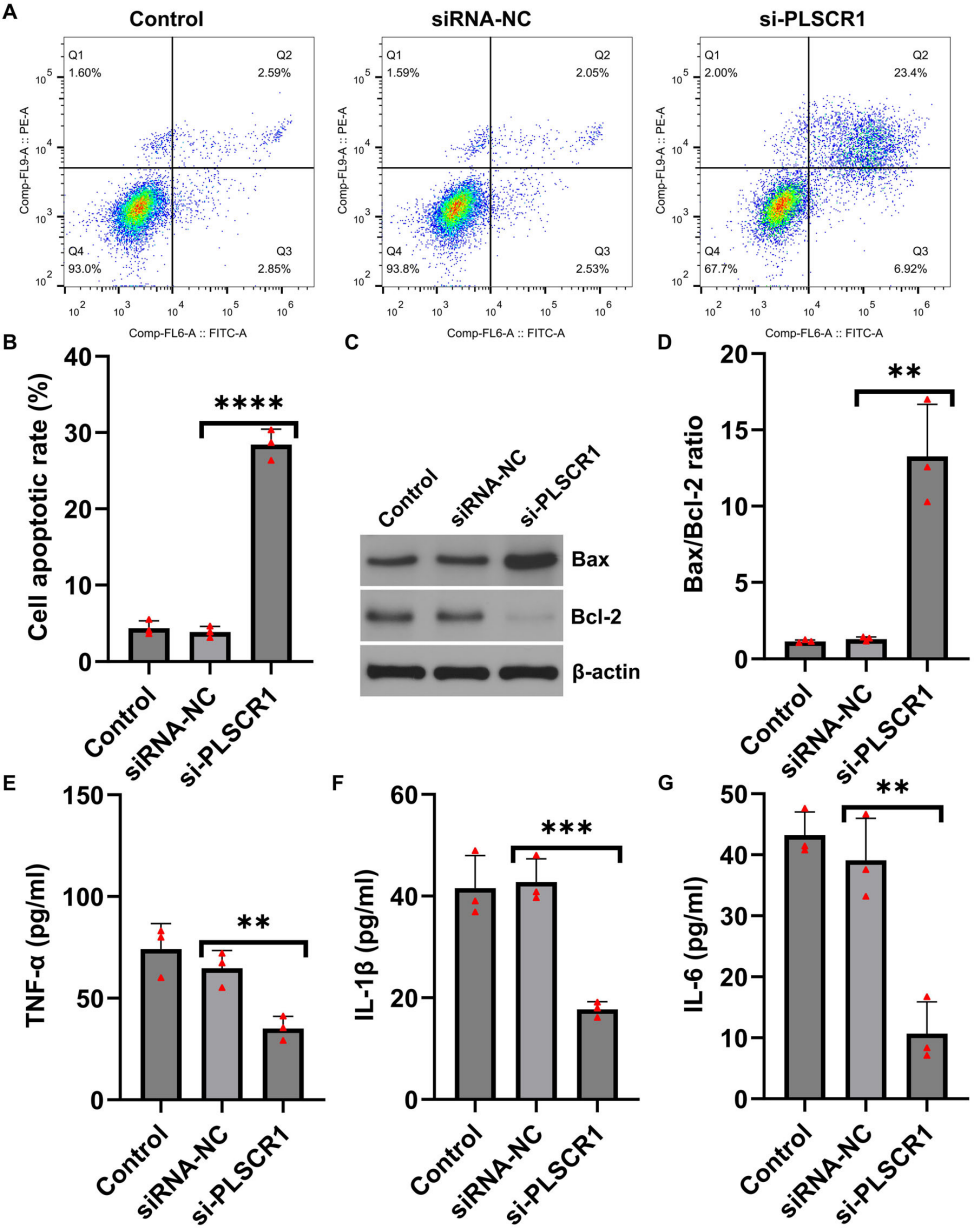
PLSCR1 regulates apoptosis and inflammation in HFLSs. (A and B) Flow cytometry detected the apoptosis in HFLSs; (C) Expression levels of Bax and Bcl‐2 proteins were analyzed by western blot analysis; (D) Bax/Bcl‐2 ratio in HFLSs; (E–G) ELISA assay detected the secretion levels of TNF‐α, IL‐1β and IL‐6. Data are presented as mean ± SD; **p < 0.01; ***p < 0.001; ****p < 0.0001.

We next examined the effects of PLSCR1 knockdown on proliferation and apoptosis of HFLS. EdU assays showed a marked decrease in EdU‐positive cells in the PLSCR1‐siRNA group, suggesting that PLSCR1 silencing significantly inhibited HFLS proliferation (Figure 2D,E). Flow cytometry analysis revealed that the apoptosis rate in the PLSCR1‐siRNA group increased compared to control‐siRNA group, indicating a strong pro‐apoptotic effect of PLSCR1 knockdown (Figure 3A,B) on HFLSs. Consistently, the results of western blot analysis showed increased expression of the pro‐apoptotic protein Bax and decreased expression of the anti‐apoptotic protein Bcl‐2, leading to a significantly elevated Bax/Bcl‐2 ratio (Figure 3C,D), further supporting the pro‐apoptotic role of PLSCR1 silencing. We then assessed the impact of PLSCR1 knockdown on inflammatory cytokines production. ELISA results demonstrated that the secretion levels of TNF‐α, IL‐1β, and IL‐6 were significantly reduced in the PLSCR1‐siRNA group (Figure 3E–G), indicating that PLSCR1 was be involved in promoting inflammatory responses in HFLSs.

**Figure 4:**
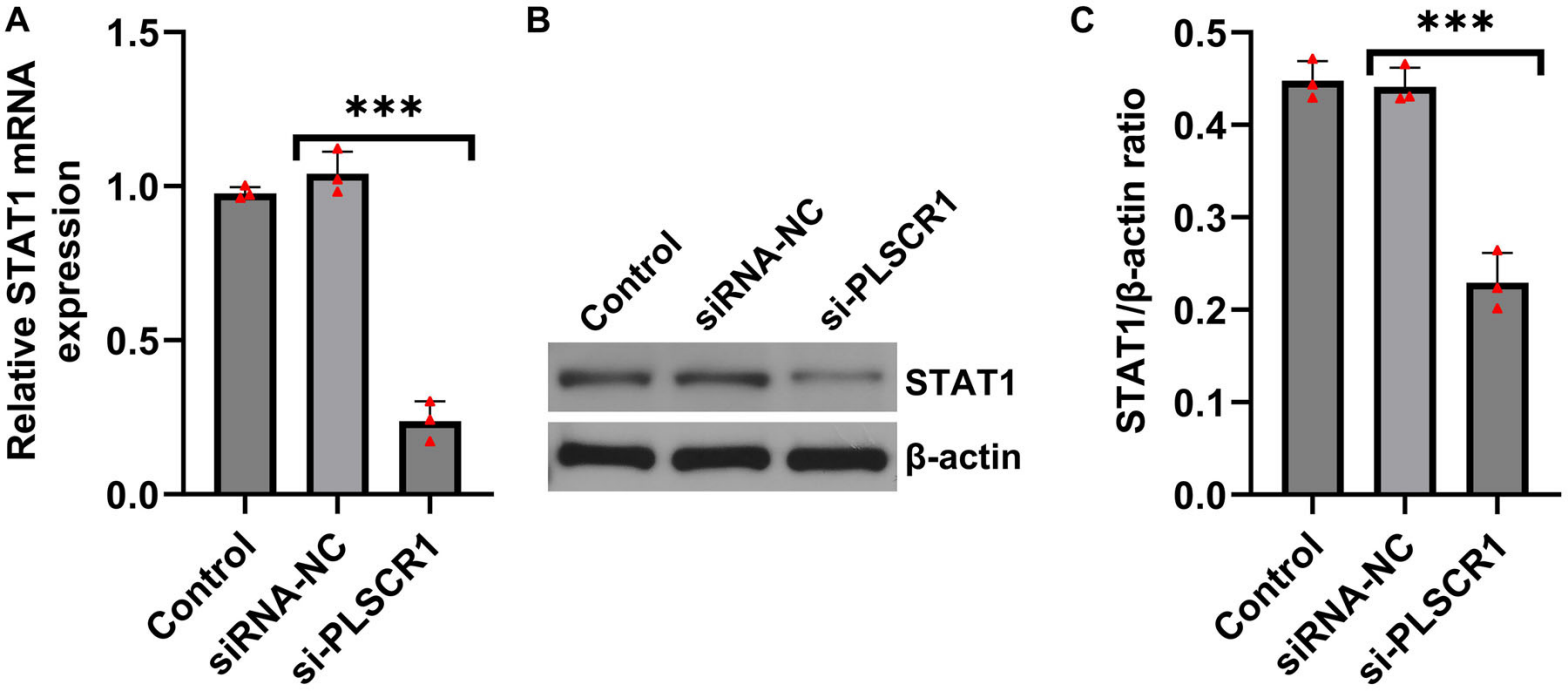
PLSCR1 regulates STAT1 expression in HFLSs. (A) The mRNA level of STAT1 in HFLSs detected by RT‐qPCR; (B and C) The protein level of STAT1 in HFLSs measured by western blot analysis. Data are presented as mean ± SD; ***p < 0.001.

Previous studies have reported that PLSCR1 can activate the STAT1 signaling pathway in breast cancer cells [24], suggesting a potential regulatory relationship. To determine whether a similar mechanism exists in RA, we assessed STAT1 expression following PLSCR1 silencing in HFLSs. RT‐qPCR and western blot analysis revealed that mRNA and protein levels of STAT1 were significantly decreased upon PLSCR1 knockdown (Figure 4A–C), suggesting that PLSCR1 positively regulated STAT1 signaling in RA.

### 3.3 PLSCR1 Modulated the Proliferation, Apoptosis, and Inflammation of HFLS via the STAT1 Signaling Pathway

To further clarify whether PLSCR1 exerted the regulatory effects on HFLSs through the STAT1 signaling pathway, we treated cells with a STAT1 enhancer, 2‐NP, in combination with PLSCR1‐siRNA and assessed changes in STAT1 expression, cell viability, apoptosis, and inflammatory cytokines production.

**Figure 5:**
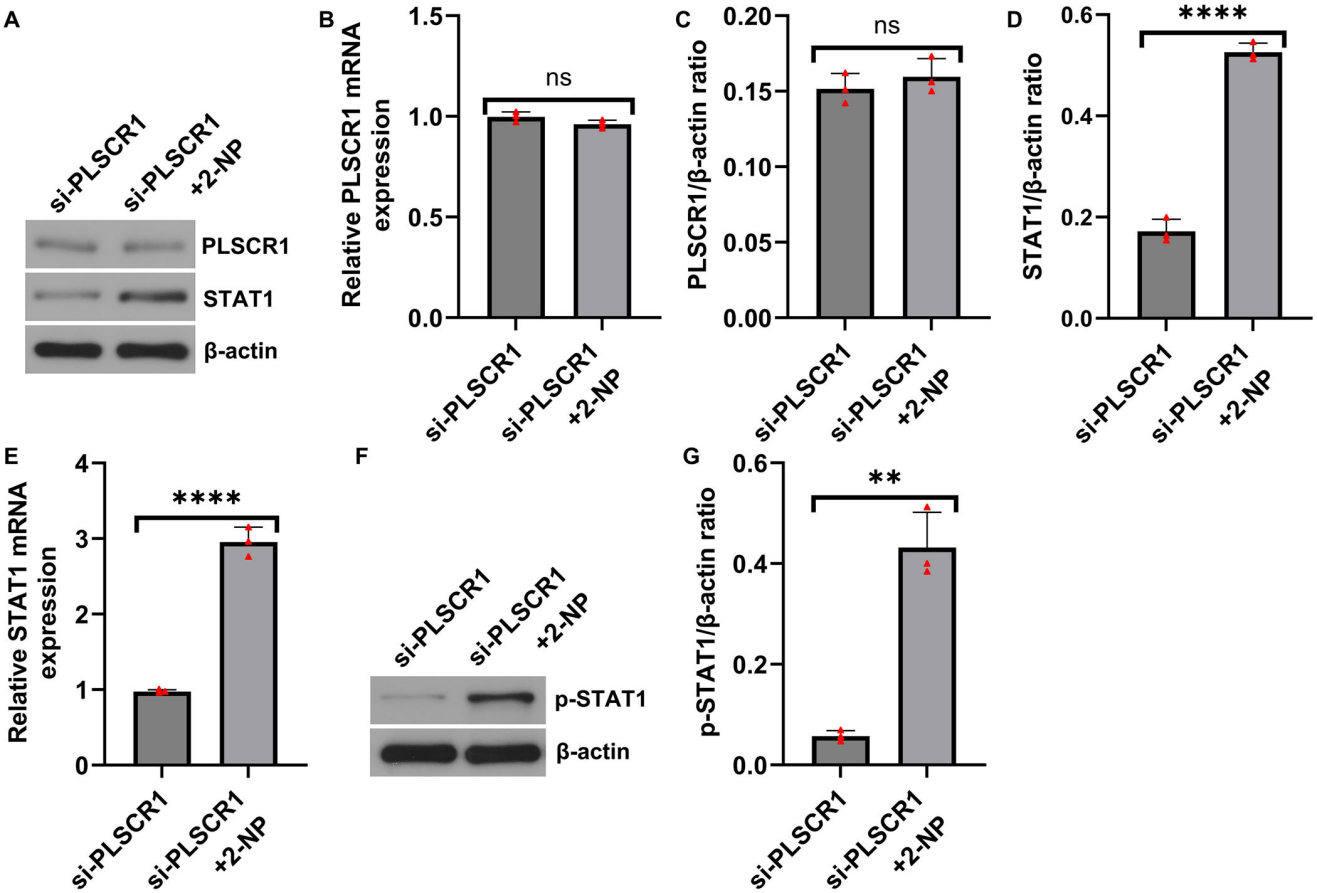
Effect of STAT1 enhancer on PLSCR1 and STAT1 expression in HFLSs. (A) The protein level of PLSCR1 and STAT1 in HFLSs measured by western blot analysi; (B) PLSCR1/β‐actin ratio; (C) STAT1/β‐actin ratio; (D and E) The mRNA level of PLSCR1 and STAT1 in HFLSs detected by RT‐qPCR; (F) The protein level of p‐STAT1 in HFLSs measured by western blot analysi; (G) p‐STAT1/β‐actin ratio. Data are presented as mean ± SD; **p < 0.01; ****p < 0.0001; ns, *p* > 0.05.

Notably, PLSCR1 expression was not restored by STAT1 activation (Figure 5A,B,D), suggesting a unidirectional regulatory relationship. RT‐qPCR and western blot analysis revealed that mRNA and protein levels of STAT1 were significantly elevated upon 2‐NP treatment in PLSCR1‐silenced HFLSs (Figure 5A,C,E), as well as enhanced p‐STAT1 expression (Figure 5F,G), indicating successful activation of the STAT1 pathway.

**Figure 6:**
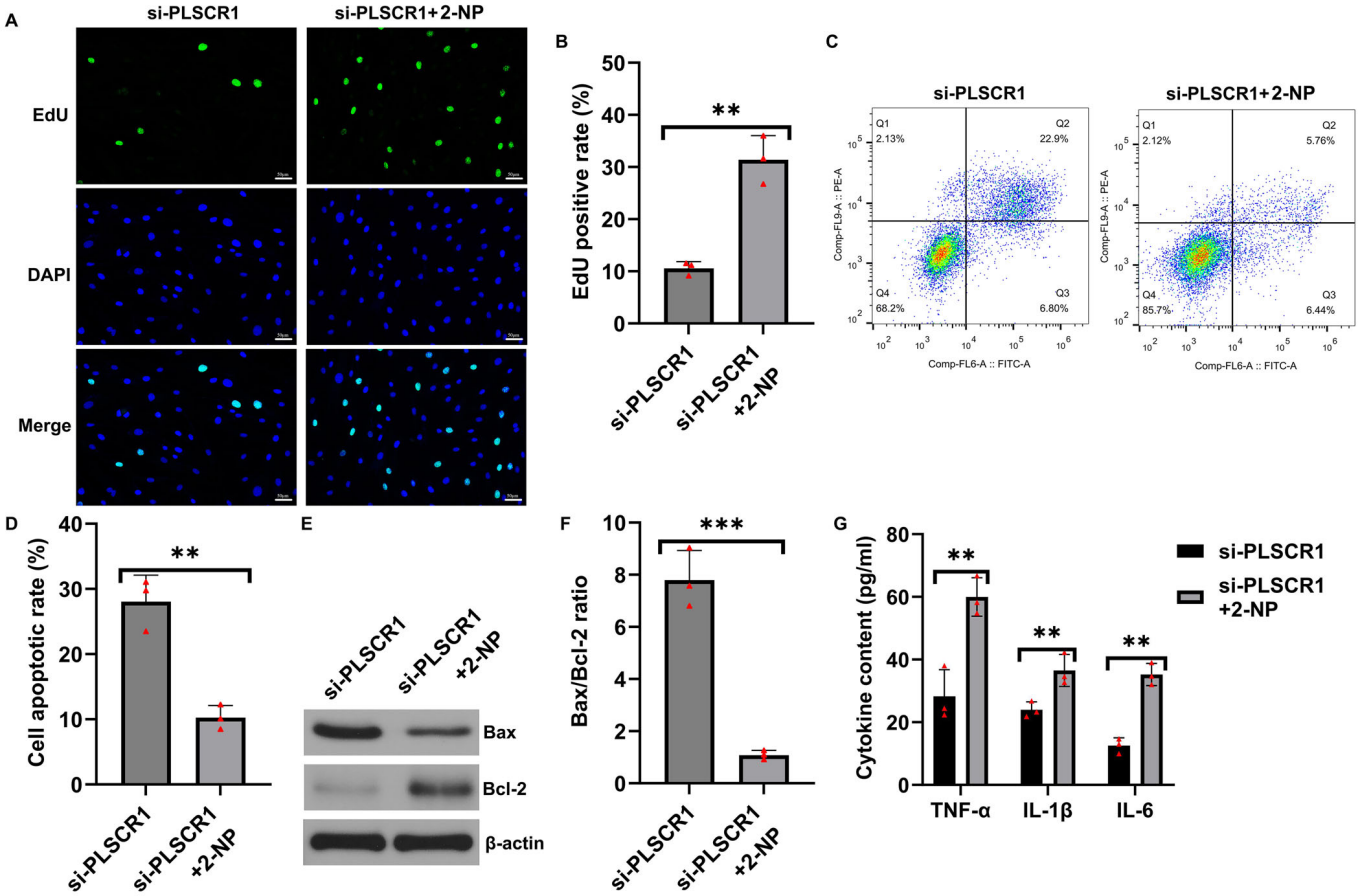
STAT1 signaling mediates the regulatory effects of PLSCR1 in HFLSs. (A and B) EdU assays showed that STAT1 activation reversed the reduced proliferation induced by PLSCR1 knockdown; (C and D) Flow cytometry detected the apoptosis in HFLSs; (E) Expression levels of Bax and Bcl‐2 proteins were analyzed by western blot analysis; (F) Bax/Bcl‐2 ratio; (G) ELISA assay detected the secretion levels of TNF‐α, IL‐1β and IL‐6. Data are presented as mean ± SD; **p < 0.01; ***p < 0.001.

Functionally, EdU assays showed that 2‐NP treatment significantly rescued the inhibitory effect of PLSCR1 knockdown on HFLS proliferation, as evidenced by a significant increase in the percentage of EdU‐positive cells (Figure 6A,B). Flow cytometry analysis demonstrated that the elevated apoptosis rate induced by PLSCR1 silencing was markedly reduced following 2‐NP treatment (Figure 6C,D). Consistently, western blot analysis results showed decreased expression of Bax and increased expression of Bcl‐2 in the combined treatment group, leading to a lower Bax/Bcl‐2 ratio in the combined treatment group compared to PLSCR1 knockdown group (Figure 6E,F), further supporting the reduction in apoptosis.

A similar trend was observed for inflammatory cytokines. Upon STAT1 activation in PLSCR1‐silenced cells, the secretion levels of TNF‐α, IL‐1β, and IL‐6 were significantly increased (Figure 6G), reversing the suppressive effect of PLSCR1 knockdown on inflammatory responses.

These results suggest that STAT1 signaling mediates the regulatory effects of PLSCR1 on HFLS proliferation, apoptosis, and inflammatory responses (Figure S1).

## 4 Discussion

In this study, we found that PLSCR1 was significantly upregulated in the serum of patients with RA. Silencing of PLSCR1 markedly inhibited the proliferation of FLSs, promoted apoptosis, and reduced the secretion of inflammatory cytokines. Further mechanistic analysis revealed that PLSCR1 regulated the biological behavior of FLSs by activating the STAT1 signaling pathway. Notably, treatment with a 2‐NP reversed the phenotypic changes induced by PLSCR1 knockdown, suggesting that the regulatory effects of PLSCR1 on FLSs were dependent on STAT1 pathway activation. These findings indicated PLSCR1 may serve as a critical modulator of FLSs activation and inflammation maintenance in RA, and could represent a potential target for therapeutic intervention.

PLSCR1 is one of the members of the PLSCR family [28]. Initially, it was identified as a calcium‐dependent membrane protein involved in the transbilayer movement of phospholipids, playing a key role in maintaining plasma membrane asymmetry [29]. In recent years, accumulating evidence has revealed that PLSCR1 is not only involved in apoptotic signaling, but also participates in a wide range of biological processes, including tumor differentiation, interferon responses, and antiviral defense [19, 30, 31]. Studies have shown that during influenza A virus infection, PLSCR1 regulates interferon lambda receptor 1 and IFN‐α signaling pathways [32]. As an ISG, PLSCR1 is markedly upregulated by IFN‐α and IFN‐γ, enhancing innate immunity and antiviral activity primarily through activation of the STAT1 signaling pathway [18]. Recent studies further demonstrate PLSCR1 can inhibit the entry of enveloped viruses such as SARS‐CoV‐2 by interfering with viral membrane fusion, a mechanism independent of the classical lipid‐scrambling function [31]. In various malignancies, PLSCR1 has been shown to promote cell survival and modulate key signaling pathways [23]. However, the role of PLSCR1 in RA remains poorly characterized. Our results suggested for the first time that PLSCR1 was not only involved in basic functions such as membrane phospholipid transport, but might also play a regulatory role in the RA synovial microenvironment by affecting intracellular inflammatory signaling pathways.

Our results indicated that the aberrant expression of PLSCR1 in HFLSs may promote a pro‐inflammatory and anti‐apoptotic phenotype by upregulating STAT1. The STAT1 signaling pathway has been widely recognized as persistently activated during RA pathogenesis, contributing to the expression of inflammatory cytokines, enhanced cell migration, and synovial tissue destruction [33, 34, 35]. In this study, we found that silencing of PLSCR1 significantly reduced both the mRNA and protein levels of STAT1, suggesting that PLSCR1 might act as an upstream regulator of the STAT1 pathway. Interestingly, activation of STAT1 effectively reversed the effects of PLSCR1 knockdown, restoring HFLSs proliferation, reducing apoptosis, and re‐elevating pro‐inflammatory cytokine secretion. These findings further supported the pivotal role of the PLSCR1–STAT1 axis in regulating HFLSs function.

Notably, PLSCR1 is also highly expressed in other chronic autoimmune diseases such as SLE [22], suggesting that it may participate in shared pathogenic mechanisms across immune dysregulation‐related disorders. In this study, we investigated the role of PLSCR1 in the synovial microenvironment of RA for the first time and established a causal link between PLSCR1 expression and the aberrant activation of FLSs. These findings provided a new perspective on the pathogenesis of RA.

However, our current conclusions are primarily based on in vitro cellular models. Further validation using in vivo models or synovial tissues derived from RA patients is necessary to confirm the functional relevance of the PLSCR1–STAT1 axis in RA progression. Additionally, it remains to be elucidated whether PLSCR1 engages in crosstalk with other key inflammatory signaling pathways, such as NF‐κB and JAK/STAT, which may further refine our understanding of the role in the inflammatory network.

In conclusion, this study was the first to reveal a novel mechanism of PLSCR1 regulating the function of HFLSs through the STAT1 signaling pathway. These findings provide new theoretical insights into the pathogenesis of synovial inflammation in RA and suggest PLSCR1 may represent a promising therapeutic target for RA intervention.

## Author Contributions

**Tianhua Chen:** conceptualization, data curation, investigation, methodology, project administration, writing – original draft, writing – review and editing. **Jiaojiao Wang:** conceptualization, supervision, writing – review and editing. All authors read and approved the final manuscript.

## Ethics Statement

All samples were obtained from the hospital with approval from the institutional ethics committee. Written informed consent was obtained from every patient.

## Consent

All patients agreed for publication.

## Conflicts of Interest

The authors declare no conflicts of interest.

## Supporting information


**Supporting Figure 1:** A schematic of the PLSCR1–STAT1 mechanism in RA.

## Data Availability

The data sets used and/or analyzed during the current study are available from the corresponding author on reasonable request.

## References

[1] M. Cross , E. Smith , D. Hoy , et al., “The Global Burden of Rheumatoid Arthritis: Estimates From the Global Burden of Disease 2010 Study,” Annals of the Rheumatic Diseases 73, no. 7 (2014): 1316–1322. 24550173 10.1136/annrheumdis-2013-204627

[2] I. B. McInnes and G. Schett , “The Pathogenesis of Rheumatoid Arthritis,” New England Journal of Medicine 365, no. 23 (2011): 2205–2219, 10.1056/NEJMra1004965. 22150039

[3] E. Mysler , M. Caubet , and A. Lizarraga , “Current and Emerging DMARDs for the Treatment of Rheumatoid Arthritis,” Open Access Rheumatology: Research and Reviews 13 (2021): 139–152, 10.2147/OARRR.S282627. 34104009 PMC8179789

[4] G. S. Firestein , “Evolving Concepts of Rheumatoid Arthritis,” Nature 423, no. 6937 (2003): 356–361, 10.1038/nature01661. 12748655

[5] S. Rantapää‐Dahlqvist , B. A. De Jong , E. Berglin , et al., “Antibodies Against Cyclic Citrullinated Peptide and IgA Rheumatoid Factor Predict the Development of Rheumatoid Arthritis,” Arthritis & Rheumatism 48, no. 10 (2003): 2741–2749. 14558078 10.1002/art.11223

[6] N. Bottini and G. S. Firestein , “Duality of Fibroblast‐Like Synoviocytes in RA: Passive Responders and Imprinted Aggressors,” Nature Reviews Rheumatology 9, no. 1 (2013): 24–33, 10.1038/nrrheum.2012.190. 23147896 PMC3970924

[7] G. Nygaard and G. S. Firestein , “Restoring Synovial Homeostasis in Rheumatoid Arthritis by Targeting Fibroblast‐Like Synoviocytes,” Nature Reviews Rheumatology 16, no. 6 (2020): 316–333, 10.1038/s41584-020-0413-5. 32393826 PMC7987137

[8] J. Falconer , A. N. Murphy , S. P. Young , et al., “Synovial Cell Metabolism and Chronic Inflammation in Rheumatoid Arthritis,” Arthritis & Rheumatology 70, no. 7 (2018): 984–999, 10.1002/art.40504. 29579371 PMC6019623

[9] F. Akbari‐Papkiadehi , A. A. Saboor‐Yaraghi , E. Farhadi , et al., “Effect of Curcumin on the Expression of NOD2 Receptor and Pro‐Inflammatory Cytokines in Fibroblast‐Like Synoviocytes (FLSs) of Rheumatoid Arthritis (RA) Patients,” Advances in Rheumatology 63 (2023): 27, 10.1186/s42358-023-00308-0. 37370181

[10] V. Tsaltskan and G. S. Firestein , “Targeting Fibroblast‐Like Synoviocytes in Rheumatoid Arthritis,” Current Opinion in Pharmacology 67 (2022): 102304, 10.1016/j.coph.2022.102304. 36228471 PMC9942784

[11] S. Yue , J. Fan , D. Xie , et al., “Unveiling the Therapeutic Potential: Targeting Fibroblast‐Like Synoviocytes in Rheumatoid Arthritis,” Expert Reviews in Molecular Medicine 27 (2025): e18, 10.1017/erm.2025.11. 40468839 PMC12201960

[12] R. Ganesan and M. Rasool , “Fibroblast‐Like Synoviocytes‐Dependent Effector Molecules as a Critical Mediator for Rheumatoid Arthritis: Current Status and Future Directions,” International Reviews of Immunology 36, no. 1 (2017): 20–30, 10.1080/08830185.2016.1269175. 28102734

[13] K. M. Kodigepalli , K. Bowers , A. Sharp , and M. Nanjundan , “Roles and Regulation of Phospholipid Scramblases,” FEBS Letters 589, no. 1 (2015): 3–14, 10.1016/j.febslet.2014.11.036. 25479087

[14] T. Wiedmer , Q. Zhou , D. Y. Kwoh , and P. J. Sims , “Identification of Three New Members of the Phospholipid Scramblase Gene Family,” Biochimica et Biophysica Acta (BBA) – Biomembranes 1467, no. 1 (2000): 244–253, 10.1016/s0005-2736(00)00236-4. 10930526

[15] B. Lu , P. J. Sims , T. Wiedmer , et al., “Expression of the Phospholipid Scramblase (PLSCR) Gene Family During the Acute Phase Response,” Biochimica et Biophysica Acta (BBA) – Molecular and Cell Biology of Lipids 1771, no. 9 (2007): 1177–1185, 10.1016/j.bbalip.2007.05.002. 17590392

[16] B. Dong , Q. Zhou , J. Zhao , et al., “Phospholipid Scramblase 1 Potentiates the Antiviral Activity of Interferon,” Journal of Virology 78, no. 17 (2004): 8983–8993. 15308695 10.1128/JVI.78.17.8983-8993.2004 PMC506946

[17] Q. Zhou , J. Zhao , F. Al‐Zoghaibi , et al., “Transcriptional Control of the Human Plasma Membrane Phospholipid Scramblase 1 Gene Is Mediated by Interferon‐α,” Blood, The Journal of the American Society of Hematology 95, no. 8 (2000): 2593–2599. 10753839

[18] K.‐W. Zhao , D. Li , Q. Zhao , et al., “Interferon‐α‐Induced Expression of Phospholipid Scramblase 1 Through STAT1 Requires the Sequential Activation of Protein Kinase Cδ and JNK,” Journal of Biological Chemistry 280, no. 52 (2005): 42707–42714. 16260419 10.1074/jbc.M506178200

[19] J. Luo , Q. Lian , D. Zhu , et al., “PLSCR1 Promotes Apoptosis and Clearance of Retinal Ganglion Cells in Glaucoma Pathogenesis,” Genes & Diseases 10, no. 4 (2023): 1564–1581. 37397520 10.1016/j.gendis.2022.05.036 PMC10311034

[20] C. Herate , G. Ramdani , N. J. Grant , et al., “Phospholipid Scramblase 1 Modulates FcR‐Mediated Phagocytosis in Differentiated Macrophages,” PLoS One 11, no. 1 (2016): e0145617. 26745724 10.1371/journal.pone.0145617 PMC4712888

[21] M. Caputo , “Molecular Mechanism of Synaptic Vesicles Recycling: Role of the Phospholipid Scramblase‐1 (PLSCR1)” (Université de Strasbourg, 2022).

[22] E. Suzuki , O. Amengual , T. Atsumi , et al., “Increased Expression of Phospholipid Scramblase 1 in Monocytes From Patients With Systemic Lupus Erythematosus,” Journal of Rheumatology 37, no. 8 (2010): 1639–1645, 10.3899/jrheum.091420. 20516018

[23] S. Zhou , J. Xu , and Y. Zhu , “Phospholipid Scramblase 1 Acts Through the IL‐6/JAK/STAT3 Pathway to Promote the Malignant Progression of Glioma,” Archives of Biochemistry and Biophysics 756 (2024): 110002. 38636689 10.1016/j.abb.2024.110002

[24] P. Huang , R. Liao , X. Chen , et al., “Nuclear Translocation of PLSCR1 Activates STAT1 Signaling in Basal‐Like Breast Cancer,” Theranostics 10, no. 10 (2020): 4644. 32292520 10.7150/thno.43150 PMC7150476

[25] C. J. Malemud , “The Role of the JAK/STAT Signal Pathway in Rheumatoid Arthritis,” Therapeutic Advances in Musculoskeletal Disease 10, no. 5–6 (2018): 117–127. 29942363 10.1177/1759720X18776224 PMC6009092

[26] M. Lao , M. Shi , Y. Zou , et al., “Protein Inhibitor of Activated STAT3 Regulates Migration, Invasion, and Activation of Fibroblast‐Like Synoviocytes in Rheumatoid Arthritis,” Journal of Immunology 196, no. 2 (2016): 596–606, 10.4049/jimmunol.1403254. 26667168

[27] Collaborative Initiative, “2010 Rheumatoid Arthritis Classification Criteria,” Arthritis & Rheumatism 62, no. 9 (2010): 2569–2581. 20872595 10.1002/art.27584

[28] J. Dal Col , M. J. Lamberti , A. Nigro , et al., “Phospholipid Scramblase 1: A Protein With Multiple Functions via Multiple Molecular Interactors,” Cell Communication and Signaling 20, no. 1 (2022): 78, 10.1186/s12964-022-00895-3. 35650588 PMC9158361

[29] N. Arashiki , M. Saito , I. Koshino , et al., “An Unrecognized Function of Cholesterol: Regulating the Mechanism Controlling Membrane Phospholipid Asymmetry,” Biochemistry 55, no. 25 (2016): 3504–3513. 27267274 10.1021/acs.biochem.6b00407 PMC5288641

[30] H. Sadanari , M. Takemoto , T. Ishida , et al., “The Interferon‐Inducible Human PLSCR1 Protein Is a Restriction Factor of Human Cytomegalovirus,” Microbiology Spectrum 10, no. 1 (2022): e01342‐21. 35138119 10.1128/spectrum.01342-21 PMC8826943

[31] D. Xu , W. Jiang , L. Wu , et al., “PLSCR1 Is a Cell‐Autonomous Defence Factor Against SARS‐CoV‐2 Infection,” Nature 619, no. 7971 (2023): 819–827. 37438530 10.1038/s41586-023-06322-y PMC10371867

[32] A. X. Yang , L. Ramos‐Rodriguez , P. Sorkhdini , et al., “Phospholipid Scramblase 1 (PLSCR1) Regulates Interferon‐Lambda Receptor 1 (IFN‐λR1) and IFN‐λ Signaling in Influenza A Virus (IAV) Infection,” bioRxiv (2025), 10.1101/2024.11.20.624469. PMC12736948 41439508

[33] P. Kasperkovitz , N. Verbeet , T. Smeets , et al., “Activation of the STAT1 Pathway in Rheumatoid Arthritis,” Annals of the Rheumatic Diseases 63, no. 3 (2004): 233–239. 14962955 10.1136/ard.2003.013276 PMC1754903

[34] S. Wang , L. Wang , C. Wu , S. Sun , and J.‐H. Pan , “E2F2 Directly Regulates the STAT1 and PI3K/AKT/NF‐κB Pathways to Exacerbate the Inflammatory Phenotype in Rheumatoid Arthritis Synovial Fibroblasts and Mouse Embryonic Fibroblasts,” Arthritis Research & Therapy 20 (2018): 1–14. 30286793 10.1186/s13075-018-1713-x PMC6235203

[35] A. Yokota , M. Narazaki , Y. Shima , et al., “Preferential and Persistent Activation of the STAT1 Pathway in Rheumatoid Synovial Fluid Cells,” Journal of Rheumatology 28, no. 9 (2001): 1952–1959. 11550959

