# Supplementary figures and images for: PLSCR1 Regulates the Physiology of Fibroblast‐Like Synoviocytes via Modulating the STAT1 Signaling Pathway
Source: Immun Inflamm Dis. 2025 Oct 27;13(10):e70294. doi: 10.1002/iid3.70294 (PMC12558900; doi:10.1002/iid3.70294)

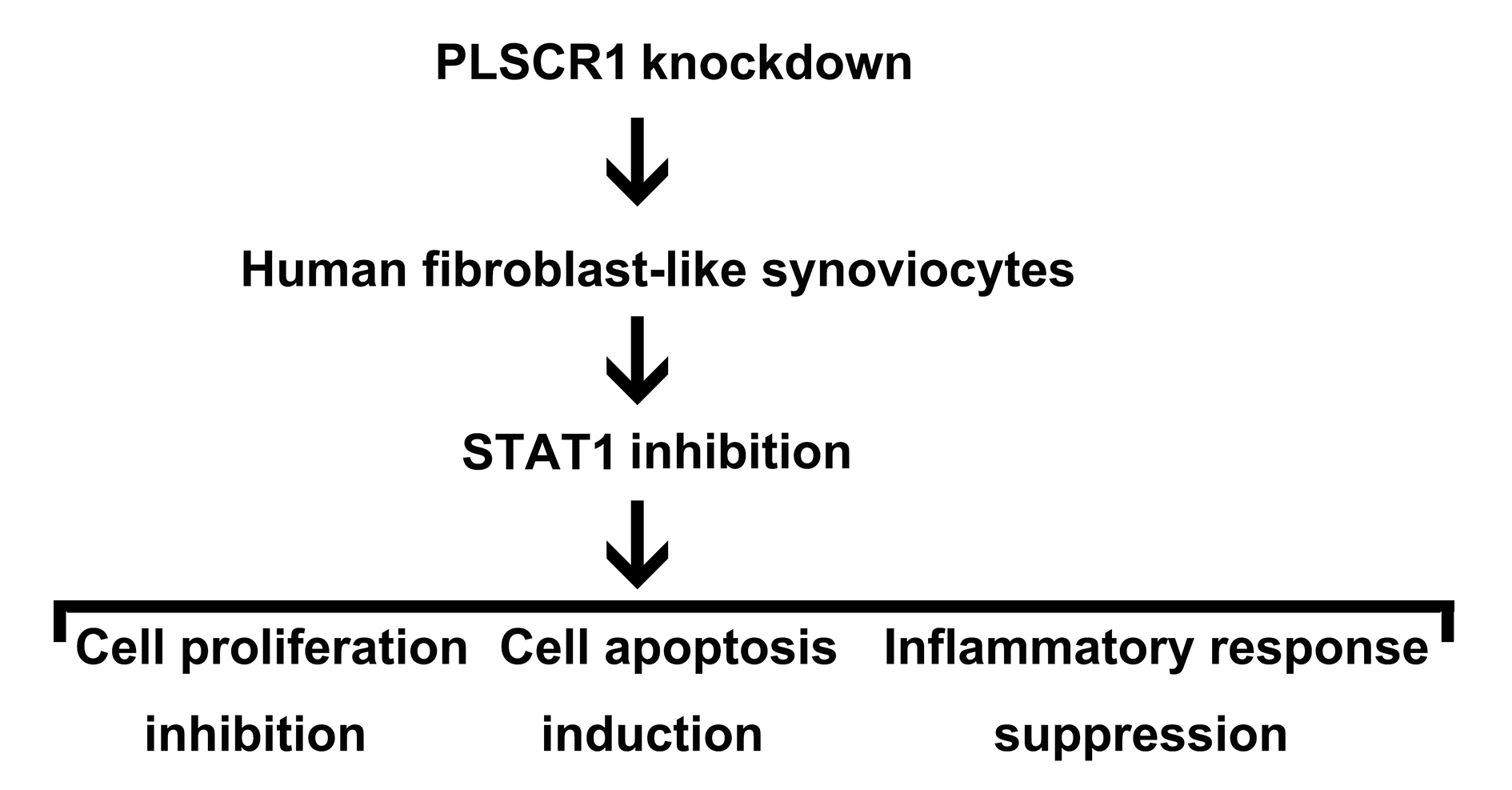

Supplement: Supplementary file 1 — Supporting Figure 1: A schematic of the PLSCR1–STAT1 mechanism in RA. [file IID3-13-e70294-s001.docx]
